# Multiple sclerosis severity variant in *DYSF-ZNF638* locus associates with neuronal loss and inflammation

**DOI:** 10.1016/j.isci.2025.112430

**Published:** 2025-04-15

**Authors:** Hendrik J. Engelenburg, Aletta M.R. van den Bosch, J.Q. Alida Chen, Cheng-Chih Hsiao, Marie-José Melief, Adil Harroud, Inge Huitinga, Jörg Hamann, Joost Smolders

**Affiliations:** 1 Neuroimmunology Research Group, Netherlands Institute for Neuroscience, 1105 BA Amsterdam, the Netherlands; 2 MS Center ErasMS, Departments of Neurology and Immunology, Erasmus MC, University Medical Center Rotterdam, 3015 CN Rotterdam, the Netherlands; 3 The Neuro (Montreal Neurological Institute-Hospital), Montréal, QC H3A 2B4, Canada; 4 Department of Neurology and Neurosurgery, McGill University, Montréal, QC H3A 2B4, Canada; 5 Department of Human Genetics, McGill University, Montréal, QC H3A 2B4, Canada; 6 Swammerdam Institute for Life Sciences, University of Amsterdam, 1054 BE Amsterdam, the Netherlands; 7 Department of Experimental Immunology, Amsterdam institute for Immunology and Infectious Diseases, Amsterdam University Medical Center, 1105 AZ Amsterdam, the Netherlands

**Keywords:** Neurology, Genetics, Immunology

## Abstract

The genetic variant rs10191329^AA^ has been identified to associate with faster disability accrual in multiple sclerosis (MS). We investigated the impact of rs10191329^AA^ carriership on MS pathology and flanking genes dysferlin (*DYSF*) and zinc finger protein 638 (*ZNF638*) in the Netherlands Brain Bank cohort (*n* = 290) by comparing rs10191329^AA^ (*n* = 6) to matched rs10191329^CC^ carriers (*n* = 12). rs10191329^AA^ carriership associated with more acute axonal stress, reduced layer 2 neuronal density, and a higher proportion of lesions with foamy microglia. In rs10191329^AA^ donors, normal appearing white matter was characterized by a higher proportion of ZNF638^+^ oligodendrocytes, and normal appearing gray matter showed more DYSF^+^ cells. Nuclear RNA sequencing showed an upregulation of mitochondrial genes in rs10191329^AA^ carriers. These data suggest that MS severity associates with an increased susceptibility to neurodegeneration and chronic inflammation. Understanding the role of DYSF, ZNF638, and mitochondrial pathways may reveal new therapeutic targets to attenuate MS progression.

## Highlights

• MS lesions of rs10191329^AA^ donors present more often with foamy myeloid cells
• Prevalence of neuro-axonal damage is increased in rs10191329^AA^ MS donors
• Neurons and oligodendrocytes of rs10191329^AA^ MS donors exhibit mitochondrial stress

## Graphical abstract

**Figure:**
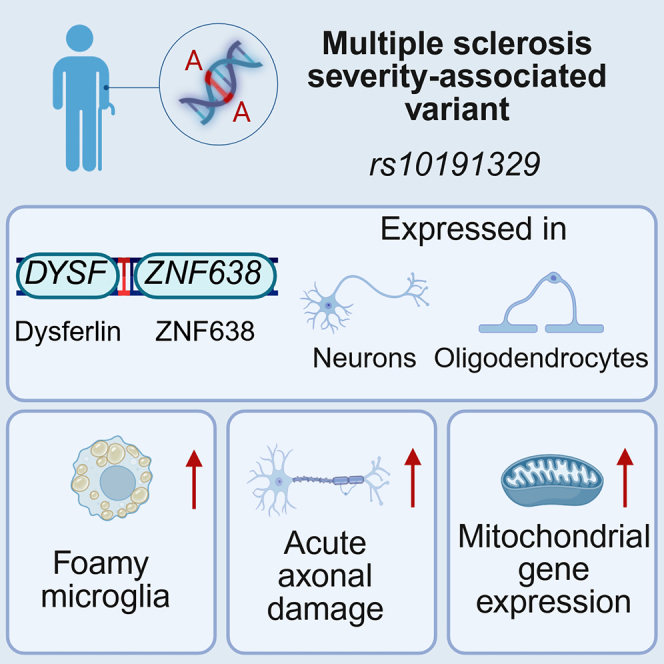

## Introduction

Multiple sclerosis (MS) is an inflammatory disease of the central nervous system (CNS). Clinically, MS is characterized by progressive accumulation of neurological disability together with inflammatory attacks resulting in neurological deficits which may be transient or can contribute to disability accumulation.^1^ Pathologically, MS is partly characterized by focal, demyelinating lesions throughout the CNS.^2^ These lesions typically display neuroinflammatory characteristics, such as activated microglia/macrophages and the presence of (resident) lymphocytes.^2^^,^^3^^,^^4^^,^^5^ MS lesions may also display neurodegenerative components,^6^ such as neuro-axonal loss and acute axonal stress, the extent of which is associated with the presence of myelin laden foamy microglia.^7^

To date, over 200 genetic variants have been associated with increased susceptibility for MS. Most of these associated loci are related to immunological pathways.^8^ In line with this, immune-modulating and -depleting therapies are efficacious in preventing early attacks of MS. However, especially in advanced disease, there is an unmet need for therapies capable of halting and reversing progression of the disease due to limited knowledge of underlying neurodegenerative processes driving disability progression.^9^

Recently, a genetic locus was reported that affects disease severity. A genome-wide association study (GWAS) of 12,584 people with MS identified homozygous carriership of the A allele at variant rs10191329 in the *DYSF*-*ZNF638* locus, hereafter referred to as rs10191329^AA^, to associate with a 3.7-year shorter median time to require a walking aid. Homozygous carriers in the MS cohort of the Netherlands Brain Bank (NBB) showed a more extensive demyelinating disease as evidenced by an increased brainstem lesion load and cortical lesion rate.^10^ This indicates the much closer distance between genetics and pathology as compared to clinical phenotype and provided early validation that the association of rs10191329 with MS severity is biologically relevant. This is reinforced by earlier investigation which showed that people with rs10191329^AA^ also presented with increased brain atrophy on magnetic resonance imaging (MRI).^11^ Importantly, a better understanding of the biological implications of this variant on MS pathology could reveal essential underlying mechanisms of therapy-resistant disease progression in MS. Here, we analyzed the NBB MS cohort using immunohistochemistry and gene expression analysis to further characterize the pathological features associated with rs10191329 and potentially uncover biological mechanisms driving disease progression.

## Results

### rs10191329^AA^ variant associates with foamy microglia morphology

Within the NBB MS autopsy cohort (*n* = 290), we identified 6 (2.1%) homozygous rs10191329 A allele carriers, which is in line with a reported minor allele frequency of 0.17 in people with MS.^10^ Clinical and pathological characteristics between rs10191329^AA^ carriers, homozygous non-carriers (hereafter rs10191329^CC^), and heterozygous NBB-MS donors are presented in Table 1. Although not significant, point estimates were consistently suggesting a more severe disease course in rs10191329^AA^ donors compared to all other donors, including on average a 6.16-year younger age of death (*p* = 0.22), a 9.11-year shorter disease duration (*p* = 0.17), a 6.18-year shorter period from onset of symptoms to reach expanded disability status scale (EDSS)6 (*p* = 0.18), and a 1.78-year shorter period from EDSS6 to EDSS8 (*p* = 0.42).

**Table 1:** Donor demographics, clinical characteristics, and pathological characteristics of variant rs10191329 in the Netherlands Brain Bank MS cohort

	C:C	A:C	A:A	*p*-value
**Donor demographics (C:C *n* = 155, A:C *n* = 68, A:A *n* = 6)**				

Age (years)	62.83 (12.75)	64.63 (13.62)	56.67 (12.03)	0.22
Sex (F/M)	97/58	43/25	6/0	0.15
PMD (hours)	8:38 (2:45)	7:45 (2:08)	9:36 (1:03)	0.25
pH of CSF	6.47 (0.29)	6.52 (0.35)	6.50 (0.48)	0.92
Weight of brain (g)	1183.76 (8.05)	1193.65 (130.14)	1138.60 (119.72)	0.94

**Clinical characteristics (C:C *n* = 155, A:C *n* = 68, A:A *n* = 5)**				

MS type (R/PP/SP)	10/47/95	4/20/38	0/1/5	0.45
Duration of disease (years)	30.44 (16.53)	31.86 (14.94)	21.33 (10.76)	0.17
Age at onset (years)	28.54 (19.57)	31.36 (19.68)	35.33 (7.87)	0.81
Years from onset to EDSS6	15.38 (11.62)	16.61 (12.70)	9.20 (5.63)	0.18
Years from EDSS6 to EDSS8	6.78 (5.26)	8.38 (6.64)	5.00 (2.45)	0.42

**Pathological characteristics (C:C *n* = 146, A:C *n* = 64, A:A *n* = 6)**				

Lesion load	13.29 (11.55)	10.29 (8.42)	21.67 (10.84)	0.01
Reactive site load	2.14 (3.30)	3.12 (6.92)	2.17 (2.71)	0.97
Proportion active	0.20 (0.21)	0.20 (0.22)	0.26 (0.20)	0.63
Proportion mixed	0.29 (0.24)	0.28 (0.26)	0.40 (0.22)	0.20
Proportion active & mixed with ramified microglia	0.23 (0.23)	0.21 (0.18)	0.17 (0.12)	0.01
Proportion active & mixed with foamy microglia	0.12 (0.18)	0.13 (0.20)	0.29 (0.25)	0.04
Proportion inactive	0.32 (0.28)	0.32 (0.27)	0.26 (0.18)	0.46
Proportion remyelinated	0.32 (0.24)	0.32 (0.27)	0.20 (0.18)	0.29
Cortical lesion rate	12.17 (16.80)	9.11 (12.71)	49.67 (40.69)	0.003

F, female; M, male; PP, primary progressive; R, relapsing; SP, secondary progressive. Significance tested between rs10191329^CC^ and rs10191329^AC/CA^ versus rs10191329^AA^ donors. Proportions were tested for significance using a quasi-binomial regression, categorical data using a chi-square test, other data using a quasi-Poisson regression.

rs10191329^AA^ donors skew toward a 1.30-times higher proportion of active (*p* = 0.63) and a 1.40-times higher proportion of mixed lesions (*p* = 0.20) compared to rs10191329^CC^ donors, although not significant. The average proportion of inactive (*p* = 0.46) and remyelinated (*p* = 0.29) lesions were comparable between groups. Most prominently, there was a 2.42-times higher abundance of active and mixed lesions with foamy microglia/macrophages in rs10191329^AA^ donors (*p* = 0.04) and, correspondingly, a 1.35-times lower proportion of lesions with ramified microglia/macrophages morphology (*p* = 0.01). In accordance with a more severe clinical disease and more extensive pathology earlier associated with rs10191329, these results indicate that there is more extensive phagocytosis by myeloid cells in carriers with a rs10191329^AA^ genotype.

### Increased neuro-axonal damage and lymphocyte infiltration in rs10191329^AA^ donors

**Figure 1:**
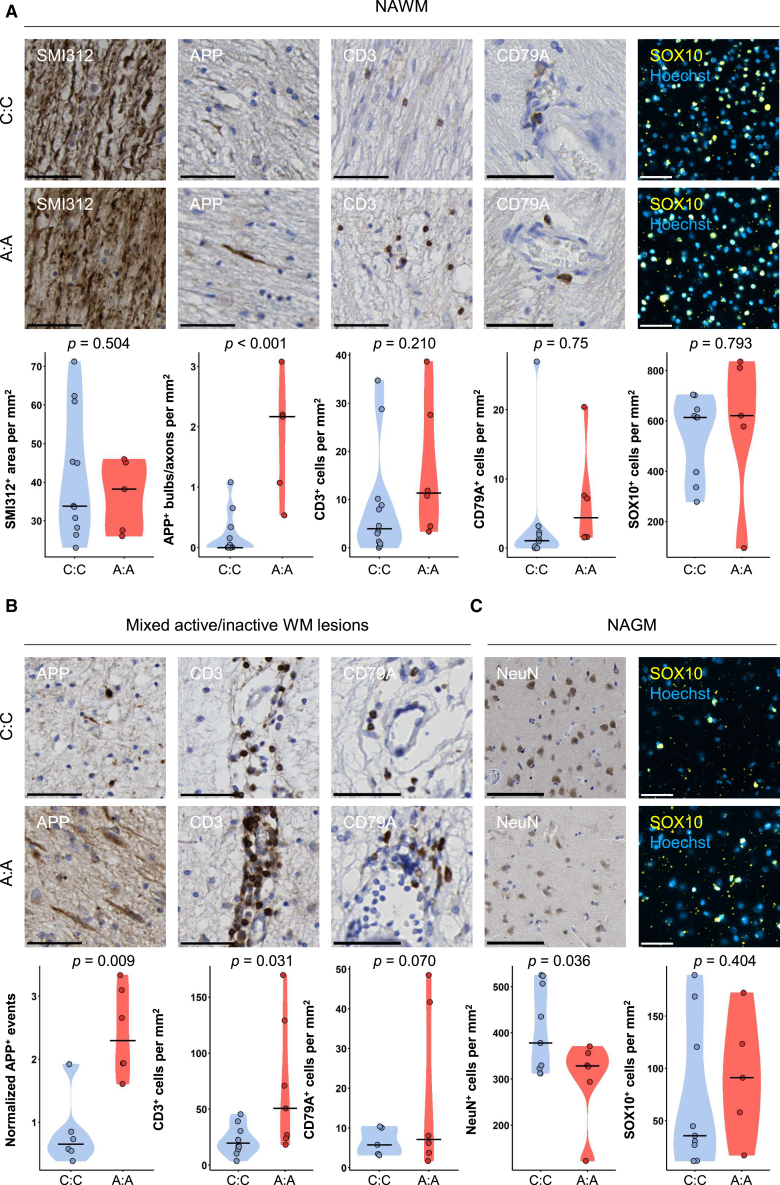
Homozygous carriers of rs10191329^AA^ have more severe neuro-axonal pathology and increased lymphocyte infiltration Panels show quantifications stratified for matched rs10191329^AA^ compared to rs10191329^CC^ donors. (A) Left to right show distribution in NAWM of axonal density as measured by surface area of SMI312 (mm^2^/mm^2^), acute axonal stress as measured by APP^+^ bulbs/axons (n/mm^2^), lymphocyte density of CD3^+^ T cells and CD79A^+^ B cells (cells/mm^2^), and density of SOX10^+^ oligodendrocytes (cells/mm^2^). Scale bars indicate 50 μm (B) Left to right show distribution in mixed lesions of acute axonal stress as measured by APP^+^ bulbs/axons normalized to NAWM (n/mm^2^), density of CD3^+^ T cells and CD79A^+^ B cells (cells/mm^2^). Scale bars indicate 50 μm (C) Left to right show distribution in NAGM of neuronal density (NeuN^+^ cells/mm^2^), and SOX10^+^ oligodendrocytes (cells/mm^2^). Scale bars indicate 100 μm and 50 μm, respectively. Data are represented as individual data points in a violin plot with a bar indicating the mean. SMI312 stainings were tested for significance with a quasi-binomial regression. Quantifications in mixed lesions were tested for significance using a mixed generalized model with a quasi-Poisson distribution. Other quantifications were tested for significance with a quasi-Poisson regression. *p* values are shown in the plots. See also Figure S1.

We next further characterized the impact of rs10191329 on MS-associated neuro-axonal damage and on lymphocyte infiltration in normal appearing white matter (NAWM) in rs10191329^AA^ and matched rs10191329^CC^ donors (Figure 1A). Although axonal density, measured with neurofilament (SMI312), was comparable (*p* = 0.50), there was a higher frequency of amyloid precursor protein (APP)^+^ bulbs/axons in pyramidal tract NAWM of rs10191329^AA^ donors compared to rs10191329^CC^ donors, reflecting acute axonal stress (*p* < 0.001). The density of CD3^+^ T cells was similar in rs10191329^AA^ and rs10191329^CC^ donors (*p* = 0.25), whereas CD79A^+^ B cell presence was increased (*p* < 0.001). Lastly, in medial-frontal gyrus NAWM, the density of SOX10^+^ oligodendrocytes was comparable between rs10191329^AA^ and rs10191329^CC^ donors (*p =* 0.79). Next, we compared the extent of acute axonal damage and infiltration of lymphocytes in mixed lesions (Figure 1B). Similar to NAWM, in rs10191329^AA^ compared to rs10191329^CC^ donors, mixed active/inactive (mixed) lesions displayed more normalized APP^+^ bulbs/axons (*p* = 0.01). Accordingly, although non-significant, rs10191329^AA^ donors showed a higher level of neurofilament light chain (NfL) *Z* scores in cerebrospinal fluid (CSF) samples (Figure S1A; *p* = 0.339), which was strongly positively correlated with the proportion of foamy (active and mixed) lesions (Figure S1B; R^2^ = 0.68, *p* = 0.043). There was a higher abundance of CD3^+^ T cells (*p* = 0.03), but not clearly of CD79A^+^ B cells (*p* = 0.27) in rs10191329^AA^ donors compared to rs10191329^CC^ donors, although with substantial variation. In normal appearing gray matter (NAGM) (Figure 1C), rs10191329^AA^ donors had a lower neuronal density measured with NeuN compared to rs10191329^CC^ donors, although not significant in all layers (*p =* 0.09), this was a robust observation in layer 2 specifically (*p* = 0.002). The density of oligodendrocytes measured with SOX10 was comparable (*p* = 0.40). Taken together, rs10191329^AA^ donors had a higher propensity of neuro-axonal damage in NAWM, mixed lesions and NAGM. This is in line with the higher lesion rate and higher abundance of foamy myeloid cells in lesions.

### Flanking regions of rs10191329 associate with oligodendrocyte presence in MS

**Figure 2:**
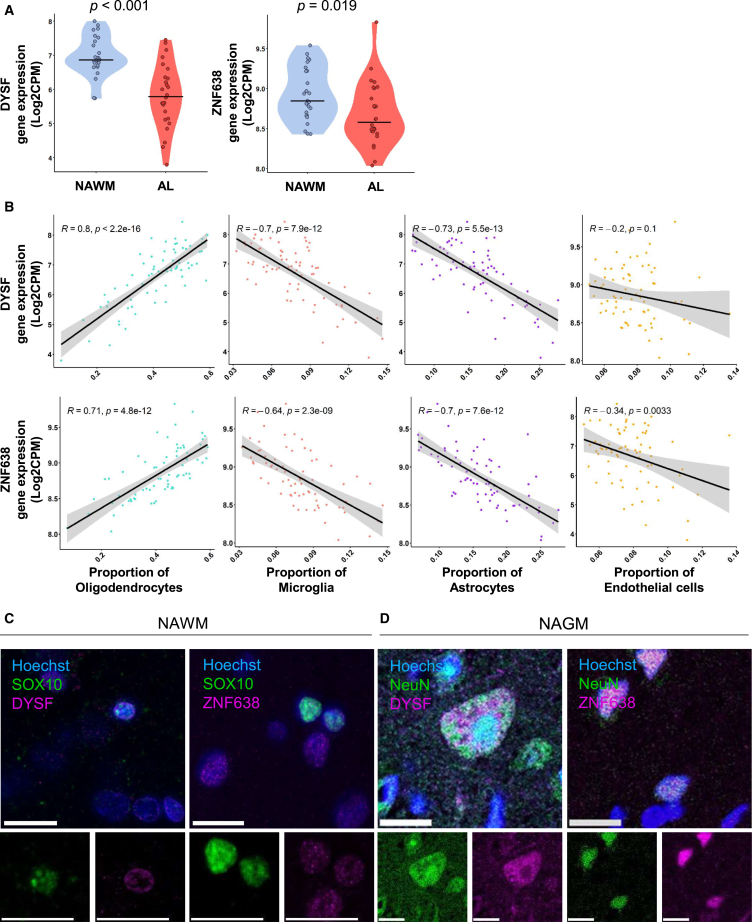
Expression of ZNF638 and DYSF in lesions and normal-appearing tissue (A) *DYSF* and *ZNF638* gene expression in active lesions compared to NAWM. Data are represented as individual data points in a violin plot with a bar indicating the mean. (B) Correlations between cell type presence assessed by cell type deconvolution and gene expression of *DYSF* and *ZNF638*. *DYSF* and *ZNF638* associates with oligodendrocytes. Data are represented as individual data points with a linear regression line and 95% confidence interval. Data were acquired from Chen et al., Brain 2025.^13^ (C and D) Immunofluorescence image of DYSF and ZNF638 double labeled (C) with SOX10, a pan-lineage oligodendrocyte marker, in NAWM and (D) with NeuN, a neuronal cell marker, in NAGM. All scale bars indicate 15 μm. Changes in gene expression between NAWM and active lesions were tested using t tests. The relationships between gene expression and cell type deconvolution were tested using a Pearson correlation. See also Figure S2.

rs10191329 is a methylation quantitative trait locus (QTL) in the promotor region of *Dysferlin* (*DYSF*), and showed a correlation with other expression and splicing QTLs for *Zinc Finger Protein 638* (*ZNF638*).^10^^,^^12^ Thus, we explored the association of these genes and their products with MS white matter pathology.^13^ In active lesions, expression of *DYSF* and *ZNF638* was lower compared to NAWM from the same sample (*p* < 0.001 and *p* = 0.02, respectively; Figure 2A). Through deconvolution, we determined the cell composition per sequenced sample. The expression of *DYSF* and *ZNF638* strongly correlated positively with the proportion of oligodendrocytes (*DYSF:* R = 0.8, *p* < 0.001*; ZNF638:* R = 0.71, *p* < 0.001), and negatively with microglia (*DYSF*: R = −0.7, *p* < 0.001; *ZNF638*: R = −0.64, *p* < 0.001), astrocyte (*DYSF*: R = −0.73, *p* < 0.001; *ZNF638*: R = −0.7, *p* < 0.001), and endothelial cell (*DYSF*: R = −0.34, *p* = 0.003; *ZNF638*: R = −0.2, *p* = 0.1) proportion (Figure 2B). Accordingly, previous single-nucleus RNA sequencing (RNA-seq) study of post-mortem brain from MS patients and controls also showed the expression of *ZNF638* and *DYSF* to be highest in oligodendrocytes and, to a lesser extent, in neurons (Figure S2).^14^ To confirm that RNA expression translated to the protein level, we performed immunohistochemistry in NAWM and verified that SOX10+ oligodendrocytes expressed DYSF and ZNF638 proteins (Figure 2C). Additionally, in NAGM, DYSF, and ZNF638 proteins also colocalized with NeuN (Figure 2D). This confirms the protein expression of DYSF and ZNF638 in oligodendrocytes and neurons.

### Increased expression of flanking genes DYSF and ZNF683 in rs10191329^AA^ donors

**Figure 3:**
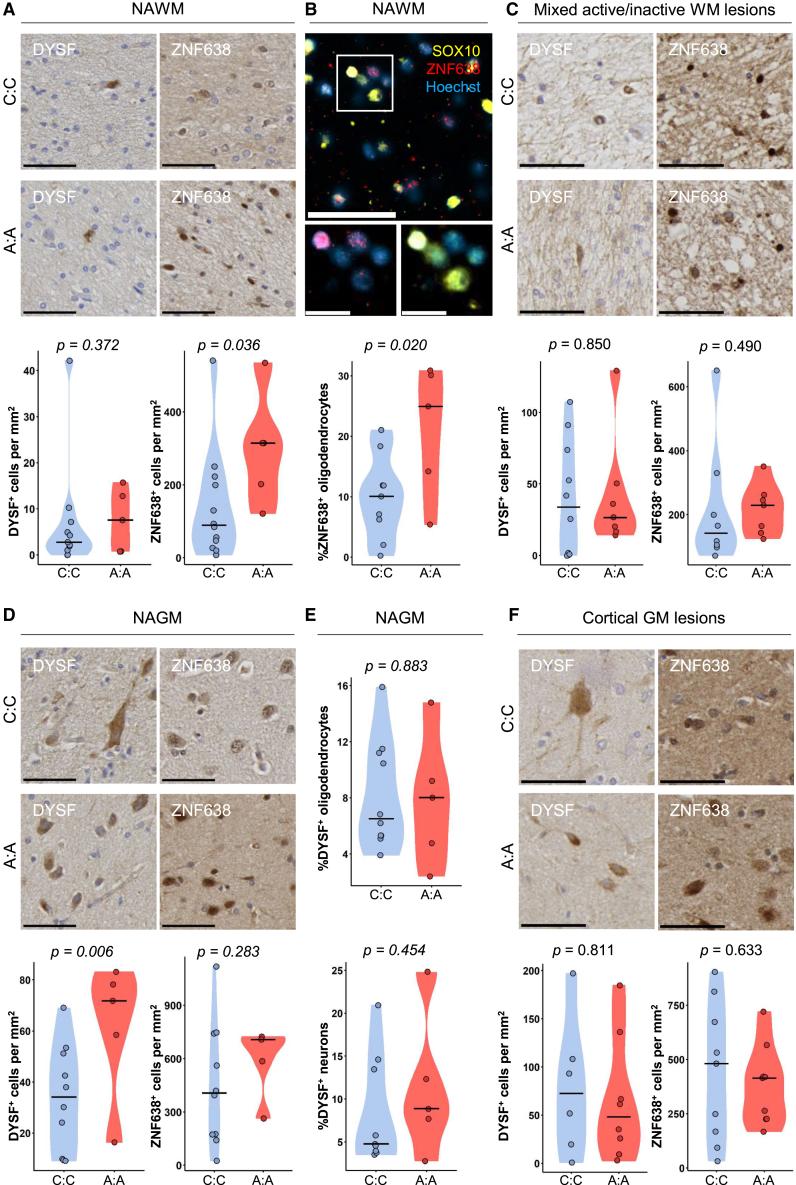
Expression of DYSF and ZNF638 by oligodendrocytes and neurons Panels show quantifications stratified for matched rs10191329^AA^ compared to rs10191329^CC^ donors. (A and B) (A) Left to right show distribution in NAWM of DYSF^+^ and ZNF638^+^ cell density (cells/mm^2^) and (B) percentage ZNF638^+^ of SOX10^+^ oligodendrocytes. Arrowhead indicates a ZNF638^+^SOX10^+^ cell. (C) Left to right show distribution in mixed lesions of DYSF^+^ and ZNF638^+^ cell density (cells/mm^2^). (D) Left to right show distribution in NAWM of DYSF^+^ and ZNF638^+^ cell density (cells/mm^2^). (E) Percentage of DYSF^+^ cells among SOX10^+^ oligodendrocytes or NeuN^+^ neurons. (F) Left to right of distribution in cortical lesions of DYSF^+^ and ZNF638^+^ cell density (cells/mm^2^). All data are represented as individual data points in a violin plot with a bar indicating the mean. Immunohistochemistry quantifications were tested for significance using a quasi-binomial generalized linear model. Scale bars of images indicate 50 μm or 15 μm (fluorescent magnifications). *p* values are shown in the plots.

Subsequently, we investigated whether there was a difference between rs10191329^AA^ and rs10191329^CC^ donors in expression of flanking genes. In NAWM, the density of cells expressing DYSF protein was comparable (*p* = 0.57). In rs10191329^AA^ compared to rs10191329^CC^ donors, we observed non-significant, but numerically higher density of ZNF638^+^ cells (*p* = 0.072; Figure 3A), which was validated by an increased ZNF638-positivity in SOX10^+^ oligodendrocytes (*p* = 0.02; Figure 3B). In mixed lesions, the density of DYSF^+^ cells and ZNF638^+^ cells were comparable between rs10191329^AA^ and rs10191329^CC^ donors (*p* = 0.85, *p* = 0.57, respectively; Figure 3C). Contrastingly, in NAGM, the density of DYSF^+^ cells was higher in rs10191329^AA^ compared to rs10191329^CC^ donors (*p* = 0.01), and the density of ZNF638^+^ cells was comparable between groups (*p* = 0.34; Figure 3D). Increased number of DYSF^+^ cells was not associated with an increased percentage of NeuN^+^ neuronal cells nor SOX10^+^ oligodendrocytes expressing DYSF (*p* = 0.88, *p* = 0.42, respectively; Figure 3E). In cortical GM lesions, the density of DYSF^+^ cells and of ZNF638^+^ cells was comparable between donors (*p* = 0.78, *p* = 0.633, respectively; Figure 3F). The altered abundance of flanking genes implicates ZNF638 and DYSF as potential molecular mediators.

### Mitochondrial changes in rs10191329^AA^ donors

**Figure 4:**
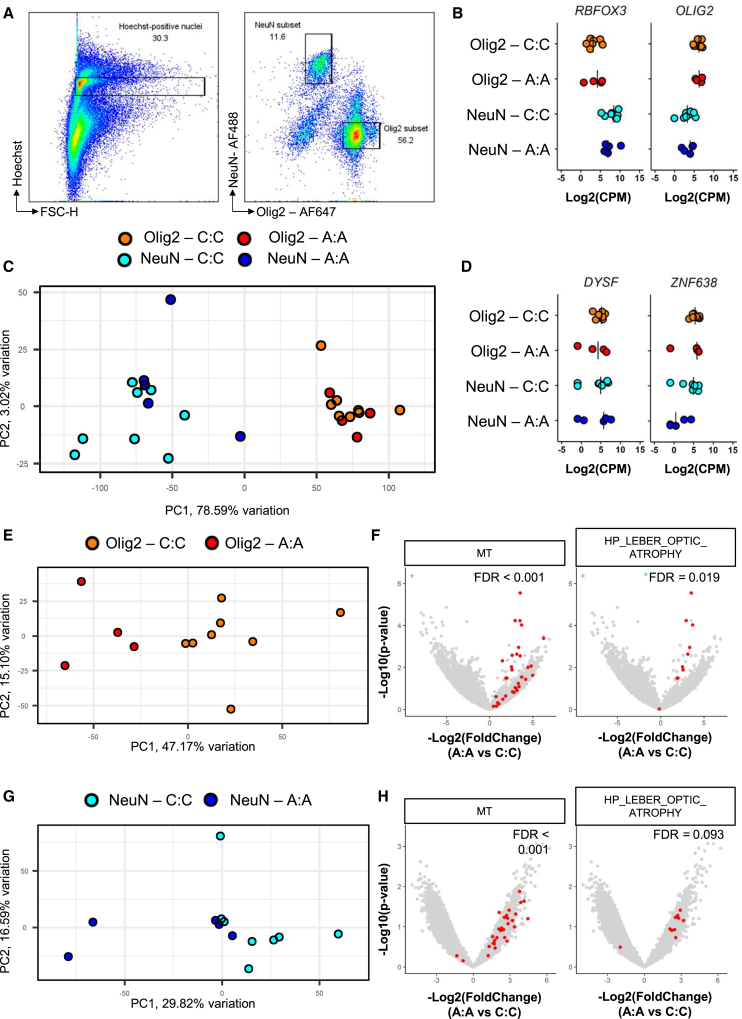
Whole transcriptome profiling of NeuN^+^ and Olig2^+^ nuclei rs10191329^AA^ and rs10191329^AA^ donors Bulk RNA-seq was performed on isolated nuclei from normal appearing superior temporal gyrus of *n* = 5 rs10191329^AA^ and *n* = 8 rs10191329^AA^ donors. (A) Representative dot plot showing flow cytometry gating strategy used for isolating nuclear subsets. Numbers indicate the percentage of events within the shown gates. (B) Expression of genes on which was gated during isolation (*RBFOX3*/NeuN and *OLIG2*). Data are represented as individual data points with a bar indicating the mean. (C) PCA plot of all samples, where the first principal component correlates with cell type. (D) Gene expression of flanking genes (*DYSF and ZNF638)* of rs10191329. Data are represented as individual data points with a bar indicating the mean. (E) PCA plot of Olig2^+^ nuclei, where the first principal component correlates with rs10191329 genotype. (F) Volcano plot of Olig2^+^ nuclei of rs10191329^AA^ versus rs10191329^CC^ donors, showing gene set enrichment analysis (GSEA) for mitochondrial genes and genes related to LHON. Points in red are part of the respective gene set. Significant genes not present in these gene sets are shown in blue. (G) PCA plot of NeuN^+^ nuclei, where the first principal component correlates with rs10191329 genotype. (H) Volcano plot of NeuN^+^ nuclei of rs10191329^AA^ vs. rs10191329^CC^ donors, showing GSEA for mitochondrial genes and genes related to Leber’s hereditary optic neuropathy. Points in red are part of the respective gene set. PCA was performed on counts-per-million that were corrected for number of nuclei input and estimated proportional cell type abundance using deconvolution. GSEA was performed using “CAMERA” from the limma R package. CPM = counts-per million; PC = principal component. See also Figure S4.

To further investigate potential driving factors of the more severe disease course, we isolated and sequenced Olig2^+^ and NeuN^+^ nuclei from normal-appearing superior temporal gyrus derived white and gray matter of rs10191329^AA^ (*n* = 5) and rs10191329^CC^ (*n* = 6) donors (Figures 4A, 4B, and S3A–S3C). Principal-component analysis (PCA) shows that the most variation was explained by cell type (Figure 4C). First, we explored expression of genes flanking rs10191329 in NeuN^+^ neuronal and Olig2^+^ oligodendrocytic nuclei, and found no apparent differences in *DYSF* and *ZNF638* transcript abundance between rs10191329^AA^ and rs10191329^CC^ donors (Figure 4D). Repeating the PCA on each nuclear subset separately, most variation was explained by genotype for rs10191329 (Figures 4E, 4G, S3D, and S3E). Yet, little significant differential gene expression between rs10191329^AA^ and rs10191329^CC^ donors was detected. In Olig2+ nuclei, *TOGARAM2* and *RMRP* were significantly downregulated in rs10191329^AA^ donors, while *MT-ND4* was upregulated (Table S1); differential gene expression analysis in NeuN+ nuclei showed no significantly differentially expressed genes after multiple testing correction. Pathway enrichment analysis showed a significant enrichment for mitochondrial genes in both Olig2^+^ (false discovery rate [FDR] <0.001) and NeuN^+^ nuclei (FDR <0.001), and specifically genes related to Leber’s hereditary optic neuropathy (LHON) in rs10191329^AA^ donors (Olig2: FDR = 0.019; NeuN: FDR = 0.093; Figures 4F, 4H, S3F, S3G, and Table S1).

## Discussion

Here, we explored the impact of rs10191329, a variant associated with MS progression, in a nested case-control study comparing homozygous rs10191329^AA^ carriers to rs10191329^CC^ carriers. After previous observation of an increased presence of focal demyelinating lesions in carriers of rs10191329^AA^,^10^ we now report (1) more immune activation in rs10191329^AA^ donors as indicated by an increased B cell presence in NAWM, and an increased frequency of foamy macrophages and T cells in mixed lesions; (2) more prominent neurodegeneration in rs10191329^AA^ donors as characterized by lower neuronal density in cortical layer 2, more acute axonal stress, and by CSF NfL levels correlating with foamy macrophage presence; (3) a positive correlation of flanking genes *DYSF* and *ZNF638* with oligodendrocyte presence in MS WM lesions; and (4) a higher percentage of oligodendrocytes expressing ZNF638 in NAWM, and a higher density of DYSF^+^ cells in NAGM in rs10191329^AA^ donors. Nuclear RNA sequencing (5) did not show any changes in *DYSF* or *ZNF638* expression but rather an upregulation of mitochondrial genes in rs10191329^AA^ donors. These data suggest that an increased susceptibility to tissue damage and inflammation could drive the association between the MS severity-associated variant rs10191329 and increased myeloid cell activation. Although its molecular mediators remain to be further studied, *DYSF*, *ZNF638*, and mitochondrial pathways are proposed as candidates affecting MS severity.

Since other studies could not replicate the association of this genetic variant to MS severity,^15^^,^^16^ possibly due to issues with power,^17^ investigating the underlying mechanisms of this genetic variant is essential. Previously, an increased abundance of foamy microglia/macrophages has been associated with increased axonal damage and acute axonal stress in MS lesions in brain donors as measured with CSF NfL chain levels.^7^ Our current replication of this observation in rs10191329^AA/CC^ donors reiterates the importance of rs10191329 as a variant relevant for MS severity. Moreover, the increase in axonal damage and decreased neuronal density in itself illustrates the skewing toward a more severe disease course in rs10191329^AA^ donors. It is known that clearance of myelin by anti-inflammatory microglia/macrophages is a prerequisite for remyelination.^18^ However, excessive myelin uptake, as is the case in foamy microglia, can be pro-inflammatory.^3^^,^^19^ A more pro-inflammatory phenotype of these brain-resident foamy phagocytes in rs10191329^AA^ donors is likely, as we found a higher lesion load, no difference in proportion of remyelinated lesions, and a positive correlation of CSF NfL levels with a higher proportion of foamy microglia/macrophages. This confirms previous reports of increased MRI brain atrophy and serum NfL-levels in living rs10191329^AA^ subjects.^11^^,^^20^ In addition, an increased amount of NAWM CD79A^+^ B cells and CD3^+^ T cells in mixed lesions of rs10191329^AA^ donors also indicates a more pro-inflammatory environment in rs10191329^AA^ donors. Taken together, rs10191329 associates with a higher propensity for tissue damage and increased immune activation.

Better understanding of the mechanism underlying this association is vital to better understand and treat disability progression in MS. Regarding the flanking genes, we have confirmed the gene and protein expression of DYSF and ZNF638 in neurons and oligodendrocytes. We also report an increase of these proteins in normal-appearing tissue of rs10191329^AA^ donors. Increased DYSF expression in NAGM could not be attributed to a specific cell type, whereas increased expression of ZNF638 in NAWM of rs10191329^AA^ donors is associated with an increase in ZNF638^+^ oligodendrocytes. These observations suggest that both molecules could be relevant to better understand the impact of rs10191329 on MS severity. The association of these molecules with neurons and oligodendrocytes primarily suggests an impact on susceptibility of these cell types to MS-inflicted tissue damage, although immune-regulatory properties have also been attributed to oligodendrocytes.^21^ We have not explored whether rs10191329 can have effects more distal than its flanking genes or other genes within the locus.

*DYSF* is well known for its expression in muscle cells, where a deficiency may cause muscular dystrophy.^22^ As a general function, DYSF is a universal mediator of membrane repair in response to damage.^23^^,^^24^^,^^25^ We do not note an alteration of DYSF expression in NAWM, but only general increased expression in NAGM without specificity for neurons. As a parallel, DYSF aggregates are seen in association with neuritic plaques in Alzheimer disease,^26^ and these aggregates are hypothesized to form due to the inability of neurons to repair membrane damage. Contrastingly, in the highly inflammatory experimental autoimmune encephalomyelitis (EAE) model of neuroinflammation, presence or absence of *DYSF* did not affect clinical course.^27^ However, this model is likely to trigger qualitatively and quantitatively different mediators of tissue damage compared to the compartmentalized inflammation as is seen in progressive MS, and may therefore not address a similar biological role of DYSF in progressive MS.

The other flanking gene, *ZNF638*, is a transcription factor implicated in transcriptional silencing in association with the human silencing hub (HUSH) complex.^28^^,^^29^ Up to now, this has been functionally described in retroviruses and adeno-associated viruses. This could be an important mechanism of action of the severity variant, especially in light of the involvement of Epstein-Barr virus (EBV) in MS pathogenesis.^30^^,^^31^ That we do not see our observed increase in protein level of ZNF638 at the transcript level make our findings less certain. Still, it has to be noted that there are a couple of factors obscuring the RNA-seq data. First, oligodendrocyte nuclei were sampled from both NAWM and NAGM, whereas an effect was only to be seen in NAWM at protein level. Furthermore, nuclear RNA mostly reflects transient and ongoing transcription and only reflects a small proportion of all RNA within a cell.^32^ Possible changes in abundance or function of ZNF638 can still indicate disease relevant consequences: (1) a higher need for viral silencing, although this would not be a logical consequence of a genetic variant or (2) a failure to silence viral DNA implicated in MS. In light of rs10191329 correlation with sQTL for *ZNF638*, this could possibly be due to alternative splicing. Our data suggest that functional differences of the variant might present in oligodendrocytes, and the mechanism of action needs further investigation.

Another possibility is that effects of rs10191329 are not a consequence of changes in abundance or function of the proximal genes of this variant. Bulk nuclear RNA sequencing suggests mitochondrial gene expression as differentiating rs10191329^AA^ and rs10191329^CC^ donors. Frequently in RNA-seq studies, high levels of mitochondrial RNA are associated with sample quality or stressed cells. Due to the randomized and balanced way in which nuclei and RNA isolations as well as sequencing were performed, and that quality metrics, such as DV200, were comparable between groups, this likely reflects a biological effect. Since we performed nuclear RNA-seq, this means that in rs10191329^AA^ donors there could be more translocation of mtRNA to the nucleus. Indeed, it has been described in endothelial cells of human diabetic donors that mtRNAs translocate to the nucleus in a cell stress- and disease-dependent manner.^33^ When mitochondrial genes are simply upregulated due to a higher abundance of mitochondria, this could indicate a higher energy demand. Alternatively, mitochondrial donation of microglia to neurons has recently been identified to occur in disease burdened neurons in order to attenuate oxidative stress.^34^ Generically higher activity of mitochondrial complex I and III can generate increased reactive oxygen species (ROS) production which is damaging to cells and tissues.^35^ As such, mitochondrial changes have been described to associate with MS pathology.^36^ For instance, changes in axonal mitochondrial frequency positively correlates with the amount of active and phagocytic microglia in the optic nerve of people with MS.^37^ Furthermore, in acute human MS lesions there are signs of focal axonal degeneration, often associated with mitochondrial pathology.^36^ In the EAE model, this was reversible by neutralization of reactive oxygen and nitrogen species.^38^ More evidence from the EAE model shows that (1) at onset of neurological symptoms, respiratory chain complex I activity is compromised in the spinal cord, and (2) mitochondrial dysfunction coincides with the presence of CD45^+^ perivascular macrophages.^39^ In line with our other findings, the increased presence of mitochondrial genes describes an worsened MS disease course in rs10191329^AA^ donors.

Of special interest is the finding that mitochondrial genes related to LHON are upregulated in rs10191329^AA^ donors. LHON is a mitochondrial genetic disease characterized by focal degeneration of the retinal ganglion cell layer and the optic nerve.^40^ In LHON, causative genetic variants cause mitochondrial dysfunction.^41^^,^^42^ The selectivity for optic nerve degeneration is hypothesized to exist because the retinal ganglion cells have the highest demand for mitochondrially produced energy.^43^ Clinically, case series of people with both MS and LHON have been published. Although the co-occurrence of these two distinct diseases has been described to likely be due to chance, MS tends to develop more severely in people with both MS and LHON.^44^ Moreover, LHON sometimes presents with an MS-like (LMS) disease with associated white matter lesions. These LMS-lesions present more often in females with LHON and are not distinguishable to MS white matter lesions on MRI.^45^ Overall, this implies a mechanistic interaction between the MS and LHON. Since mitochondrial RNA is increased in cells affected by LHON,^46^ this gene-association could indicate a similar mitochondrial dysfunction in MS, where an unmet energy demand could cause a more rapid decline in people with MS. Overall, this supports the theory that rs10191329^AA^ carriers of the severity variant would be more vulnerable to neurodegeneration coinciding with either the inability to provide enough energy to neurons or an abundance of ROS production. Whether these mitochondrial aberrancies are a cause or effect of accelerated disease course remains to be determined.

In conclusion, we show that the rs10191329 variant is associated with more extensive pathological changes in MS, such as an increased susceptibility to tissue damage and increased myeloid cell activation and disclose both DYSF and ZNF638, as well as mitochondrial pathways as potential candidates underlying these effects. In sum, we have further biologically validated the findings of the recently discovered MS-severity variant rs10191329. These findings may open new avenues for ameliorating MS progression.

### Limitations of the study

There are some limitations to this study. Due to the low minor allele frequency of 0.17 and the scarce available tissue, this study generally lacks power to investigate all components of MS pathology extensively. Nevertheless, investigation within the homogeneously sampled and processed Netherlands Brain Bank tissue collection limits confounding factors and thereby increases the sensitivity of our study. Additionally, investigating biological processes closely related to the pathophysiology of MS allows assessment of the impact of rs10191329 in a smaller sample size compared to the impact of this genetic variant on a more generic endpoint such as the age of using a walking aid. Another limitation is that the isolation of nuclei yielded low abundance of RNA, resulting in limited library sizes. Overall, the characterization as performed by the NBB^2^ and the availability of genotyped high-quality brain tissue make this dataset unique and provide insight into the association of rs10191329 with MS severity and the pathophysiology of MS.

## Resource availability

### Lead contact

Requests for further information and resources should be directed to and will be fulfilled by the lead contact, dr. Joost Smolders (j.j.f.m.smolders@erasmusmc.nl).

### Materials availability

This study did not generate new unique reagents.

### Data and code availability

Nucleus RNA-seq data deposited at GEO and are publicly available as of the date of publication. The Accession numbers of nuclei RNA sequencing is GSE273954. Tissue RNA-seq data (Fig. 2AB) is available under accession number GSE283092. This paper does not report original code. Any additional information required to reanalyze the data reported in this paper is available from the lead contact upon request.

## Acknowledgments

We are grateful to the brain donors and their families for their commitment to the Netherlands Brain Bank donor program. We are grateful to dr. Aldo Jongejan for sharing his knowledge on RNAseq analysis. Funding for this research was obtained from the Blom-de Wagt Foundation (MS Research: 23–1208), MoveS (MS Research: 17–975), and MS Research (19–1079).

## Author contributions

H.J.E., A.M.R.v.d.B., I.H., J.H., and J.S. contributed to the study concept; H.J.E., A.M.R.v.d.B., J.Q.A.C., C.-C.H., and M.-J.M. acquired and analyzed data; H.J.E. and A.M.R.v.d.B. drafted the figures; H.J.E., J.H., and J.S. verified and interpreted the underlying data; H.J.E., A.H., I.H., J.H., and J.S. wrote the manuscript; J.H. and J.S. supervised the project team; all authors reviewed and approved the manuscript for final publication.

## Declaration of interests

The authors declare no competing interests.

## STAR★Methods

### Key resources table

**Table:** 

REAGENT or RESOURCE	SOURCE	IDENTIFIER
**Biological samples**		

Human brain tissue and CSF	Netherlands Brain Bank	https://www.brainbank.nl

**Antibodies and secondary reagents**		

Rabbit-anti-human Olig2 – Alexa Fluor® 647	Abcam	Cat# ab225100 RRID: -
Donkey-anti-mouse IgG – Cy3	Jackson ImmunoResearch	Cat# 715-165-150 RRID: AB_2340813
Donkey-anti-rabbit IgG – Cy3	Jackson ImmunoResearch	Cat# 711-165-152 RRID: AB_2307443
FcR-Blocking Reagent	Miltenyi Biotec	Cat# 130-059-901 RRID: AB_2892112
Goat-anti-human SOX10	R&D Systems	Cat# AF2864 RRID: AB_442208
Horse-anti-goat-IgG – biotin	Vector Laboratories	Cat# BA-9500 RRID: AB_2336123
Horse-anti-mouse-IgG – biotin	Vector Laboratories	Cat# BA-2000 RRID: AB_2313581
Horse-anti-rabbit-IgG – biotin	Vector Laboratories	Cat# BA-1100 RRID: AB_2336201
Mouse-anti-human APP A4	Sigma-Aldrich	Cat# MAB348 RRID: AB_94882
Mouse-anti-human CD79A	DAKO	Cat# M705001-2 RRID: -
Mouse-anti-human Dysferlin	Leica	Cat# HAMLET-CE RRID: -
Mouse-anti-human NeuN	Sigma-Aldrich	Cat# MAB377 RRID: AB_2298772
Mouse-anti-human NeuN – Alexa Fluor®488	Sigma-Aldrich	Cat# MAB377x RRID: AB_2149209
Mouse-anti-human neurofilament marker	BioLegend	Cat# 837901 RRID: -
Rabbit-anti-human CD3	DAKO	Cat# A045201 RRID: -
Rabbit-anti-human ZNF638	Sigma-Aldrich	Cat# HPA036784 RRID: AB_10672478
Streptavidin – Alexa Fluor®488	Jackson ImmunoResearch	Cat# 016-540-084 RRID: AB_2337249

**Chemicals, peptides, and recombinant proteins**		

3,3′-diaminobenzidine (DAB)	DAKO	Cat# K5007
Biotinylated tyramide	Sigma-Aldrich	Cat# SML2135
Bovine serum albumin (BSA)	Sigma-Aldrich	Cat# A7906
cOmplete ULTRA tablets, Protease Inhibitor Cocktail	Merck	Cat# 5892953001
Dulbecco’s phosphate-buffered saline (DPBS)	Thermo Fisher Scientific	Cat# 14190094
Dithiothreitol (DTT)	Thermo Fisher Scientific	Cat# R0861
Entallan	Sigma-Aldrich	Cat# 1.07961
Haematoxylin	Merck	Cat# 4305
Hoechst 33342	Thermo Fisher Scientific	Cat# H3570
Hydrogen peroxide	Merck	Cat# 107209
MgCl_2_	Thermo Fisher Scientific	Cat# AM9530G
Mowiol	Merkc	Cat# 4705904
Sucrose	Sigma-Aldrich	Cat# 16104
Triton X-100	Sigma-Aldrich	Cat# X-100
UltraPure™ 0.5M EDTA, pH8.0	Thermo Fisher Scientific	Cat# 15575020
UltraPure™ 1M Tris-HCI, pH8.0	Thermo Fisher Scientific	Cat# 15568025

**Critical commercial assays**		

DNeasy Blood & Tissue Kit	Qiagen	Cat# 69506
NF-light ELISA CE kit for CSF	Uman Diagnostics	Cat# 10-7001
RNEasy micro kit	Qiagen	Cat# 74004
SMARTer Stranded Total RNA-Seq Kit v3 - Pico Input Mammalian Library Prep Kit	Takara Bio	Cat# 634485
Vector Elite ABC kit	Vector Laboratories	Cat# PK-6100 RRID: AB_2336819

**Deposited data**		

Nuclei bulk RNAseq data	This paper	GEO: GSE273954
Tissue bulk RNAseq data	Chen et al.^13^	GEO: GSE283092

**Software and algorithms**		

Application Suite X	Leica	N/A
Bioconductor (v3.13)	Huber et al.^47^	https://bioconductor.org/
biomaRt package (v.2.58.2)	Durinck et al.^48^	Bioconductor
dtangle (v.2.0.9)	Hunt et al.^49^	https://cran.r-project.org/package=dtangle
dupRadar (v1.12.1)	Sayols et al.^50^	Bioconductor
edgeR package (v3.34.1, 3.42.4)	Robinson and Oshlack^51^	Bioconductor
Emmeans package (v1.10.2)	CRAN	https://cran.r-project.org/package=emmeans
fastp (v0.23.4),	Chen et al.^52^	https://github.com/OpenGene/fastp
FastQC (v0.11.9 & v0.12.1)	Babraham Institute	https://www.bioinformatics.babraham.ac.uk/projects/fastqc/
featureCounts (v2.0.6)	Liao et al.^53^	https://subread.sourceforge.net/featureCounts.html
FlowJo (v10.10.0)	BD Biosciences	https://www.flowjo.com/
gencode.v45.primary_assembly.annotation.gtf	Gencode	https://www.gencodegenes.org/human/
ggplot2 package (v3.5.1)	Wickham^54^	https://cran.r-project.org/package=ggplot2
glmmTMB package	CRAN	https://CRAN.R-project.org/package=glmmTMB
GRCh38.105	Ensembl	https://www.ensembl.org/index.html
GRCh38.primary_assembly.genome.fa (v.45)	Gencode	https://www.gencodegenes.org/human/
HISAT2 (v2.2.1)	Kim et al.^55^	http://daehwankimlab.github.io/hisat2/
Homo_sapiens.GRCh38.105.gtf	Ensembl	https://www.ensembl.org/index.html
HTseq (v1.99.2)	Anders et al.^56^	https://htseq.readthedocs.io/en/master/
Fiji for ImageJ (v1.54f)	Schindelin et al.^57^	https://imagej.net/software/fiji/
limma package (v3.56.2)	Ritchie et al.^58^	Bioconductor
lme4 package	CRAN	https://cran.r-project.org/package=lme4
PCAtools package (v2.14.0).	N/A	Bioconductor
Qupath 0.4.0	Bankhead et al.^59^	https://qupath.github.io/
R (v4.1.0 & v4.3.1)	The R Project	https://www.r-project.org/
Samtools (v1.20)	Danecek et al.^60^	https://www.htslib.org/
STAR2 (v2.7.11b)	Dobin et al.^61^	https://github.com/alexdobin/STAR
Trimmomatic (v0.39)	Bolger et al.^62^	http://www.usadellab.org/cms/index.php?page=trimmomatic

**Other**		

Axioscan Z1	Zeiss	N/A
Ensembl (v112)	EMBL-EBI	https://www.ensembl.org/index.html
FACSAria II cell sorter	BD Bioscience	N/A
Fragment Analyzer	Advanced Analytical Technologies at GenomeScan B.V.	N/A
KASP genotyping platform	LGC Genomics	N/A
Molecular Signatures Database (MSigDB, version 2023.2)	Subramanian et al.^63^	https://www.gsea-msigdb.org/gsea/msigdb/index.jsp
NovaSeq6000	Illumina at GenomeScan B.V.	N/A
STEDYCON microscope	Abberior Instruments	N/A
TCS SP8 confocal microscope	Leica	N/A

### Experimental model and study participant details

#### Characterization of MS lesions and genotyping

Following informed consent, brain donors with pathologically confirmed MS that were recruited to the NBB since 1990 were included in this study (Table 1). Autopsy procedures were approved by the Ethics Committee of the VU medical center in Amsterdam, the Netherlands. Of all donors, detailed clinical summaries were available.

As previously described,^64^ blocks were dissected at standardized CNS locations (including the brainstem), with additional blocks targeted to MS lesions using macroscopic and post-mortem magnetic resonance imaging (MRI) assessment. Sections were stained for proteolipid protein (PLP) and human leukocyte antigen (HLA) for lesion characterization.^65^ Each MS lesion was characterized blind to genotype status. Of each donor, the reactive site load and lesion load were calculated in the standardized CNS locations, and the proportions of active, mixed lesions, inactive lesions, and remyelinated lesions, the proportion of active and mixed lesions with foamy or ramified microglia, and the cortical lesion rate were calculated as described previously.^2^ Shortly, all tissue blocks taken at autopsy were characterized for lesion type, and in case of active and mixed active/inactive lesions, the dominant microglia/macrophage morphology was registered.

#### Nested case-control cohort

For further investigation, all 6 rs10191329^AA^ donors were matched with 12 rs10191329^CC^ donors for age, sex, post-mortem delay, and pH of CSF for a nested case-control cohort (Table 2). Further, donors were selected such that differences in clinical characteristics were as small as possible, where the only characteristic approaching significance is time between EDSS6 and EDSS8. Although unfortunate, this conveys the close relationship between genotype and disease severity. As there were no clinical and cohort-pathological effects of heterozygosity for rs10191329, these donors were not included in the cohort.

**Table 2:** Donor demographics, clinical characteristics, and pathological characteristics of the nested case-control design study

	C:C (*n* = 12)	A:A (*n* = 6)	*p* value
**Donor demographics**			

Age (years)	62.33 (15.26)	56.67 (12.03)	0.41
Sex (F/M)	12/0	6/0	1.00
PMD (hours)	9:35 (3:36)	9:36 (1:03)	0.99
pH of CSF	6.32 (0.31)	6.50 (0.48)	0.46
Weight of brain (g)	1193.33 (115.19)	1138.60 (119.72)	0.41

**Clinical characteristics**			

MS type (PP/SP)	5/7	1/5	0.60
Duration of disease (years)	27.17 (9.80)	21.33 (10.76)	0.29
Age at onset (years)	35.17 (14.68)	35.33 (7.87)	0.98
Years from onset to EDSS6	12.83 (10.93)	9.20 (5.63)	0.38
Years from EDSS6 to EDSS8	10.67 (7.84)	5.00 (2.45)	0.08

**Pathological characteristics**			

Lesion load	17.58 (10.00)	21.67 (10.84)	0.46
Reactive site load	5.00 (4.31)	2.17 (2.71)	0.11
Proportion active lesions	0.30 (0.21)	0.26 (0.20)	0.70
Proportion mixed lesions	0.34 (0.21)	0.40 (0.22)	0.56
Proportion active & mixed lesions with ramified microglia	0.32 (0.16)	0.17 (0.12)	0.06
Proportion active & mixed lesions with foamy microglia	0.13 (0.12)	0.29 (0.25)	0.05
Proportion inactive lesions	0.23 (0.15)	0.26 (0.18)	0.76
Proportion remyelinated lesions	0.36 (0.20)	0.20 (0.18)	0.23
Cortical lesion rate	8.33 (11.16)	49.67 (40.69)	0.05

Proportions were tested for significance using a quasi-binomial regression, categorical data using a chi-square test, other data using a quasi-Poisson regression. F, female; M, male; PP, primary progressive; SP, secondary progressive.

Of selected donors, we collected formalin-fixed, paraffin-embedded (FFPE) tissue blocks from (1) the brainstem containing the pyramidal tract longitudinally (rs10191329^AA^, *n* = 5; rs10191329^CC^, *n* = 11), (2) mixed lesions (rs10191329^AA^, *n* = 5; rs10191329^CC^, *n* = 6) and (3) cortical lesions (rs10191329^AA^, *n* = 5; rs10191329^CC^, *n* = 5) from varying supratentorial regions, (4) NAWM (rs10191329^AA^, *n* = 5; rs10191329^CC^, *n* = 10) and NAGM (rs10191329^AA^, *n* = 5; rs10191329^CC^, *n* = 9) from a widely available standardly dissected region (the medial frontal gyrus) and (5) fresh-frozen tissue from NAWM and NAGM from another widely available standardly dissected region (the superior temporal gyrus; rs10191329^AA^, *n* = 6; rs10191329^CC^, *n* = 9).

### Method details

#### Genotyping

Of 290 MS brain donors from the NBB, DNA was extracted from whole blood or frozen cerebellar tissue using the DNeasy Blood & Tissue Kit (QIAGEN), or, alternatively, from formalin-fixed, paraffin-embedded (FFPE) cerebellar tissue. Genotyping for rs10191329 was performed using the KASP genotyping platform (LGC Genomics).

#### Quantitative immunohistochemistry

Neuronal damage, immune components, and flanking regions of rs10191329 were assessed through immunohistochemistry (IHC) on 8-μm FFPE or fresh-frozen sections. After rehydration, antigen retrieval was accomplished with microwave treatment at 700 W. Sections were incubated in 3% H_2_O_2_ in PBS for 15 minutes and subsequently incubated with a primary antibody overnight in blocking buffer (PBS + 10% normal horse serum + 1% bovine serum albumin + 0.5% TritonX) at 4°C (Table 3). Sections were incubated with appropriate biotinylated secondary antibody, followed by avidin-biotin-horseradish peroxidase (HRP) complex (Vector Elite ABC kit; Vector Laboratories), before visualization with 3,3′-diaminobenzidine (DAB) and hematoxylin counterstaining.

**Table 3:** Antibody overview for immunohistochemistry

Protein	Company (catalog #)	Host	Clone	RRID	Dilution	Fixation	Antigen retrieval
SMI312	Biolegend (837901)	Mouse	SMI312	NA	1:2,000	FFPE	CB, pH6.0
APP	Millipore (MAB348)	Mouse	A4	AB_94882	1:2,000	FFPE	CB, pH6.0
NeuN	Sigma (MAB377)	Mouse	A60	AB_2298772	1:100	FFPE	PBS, pH7.6
CD3	DAKO (A045201)	Rabbit	Polyclonal	NA	1:100 (FFPE) 1:250 (Frozen)	FFPE 4% PFA	CB, pH6.0 –
CD79A	DAKO (M705001-2)	Mouse	Polyclonal	NA	1:200	FFPE	CB, pH6.0
DYSF	Leica (HAMLET-CE)	Mouse	HAM1/7B6	NA	1:50	FFPE	CA, pH7.6
ZNF638	Sigma (HPA036784)	Rabbit	Polyclonal	AB_10672478	1:100	FFPE	CA, pH7.6
SOX10	R&D (AF2864)	Goat	Polyclonal	AB_442208	1:200	FFPE	CA, pH7.6
NeuN	Sigma (MAB377X)	Mouse	A60	AB_2149209	1:500	FFPE	CA, pH7.6

CA: citraconic anhydride; CB: citrate buffer; FFPE, formalin-fixed, paraffin-embedded; PBS, phosphate-buffered saline; PFA: paraformaldehyde; RRID, research resource identifier.

For immunofluorescence (IF) staining, sections were incubated with Cy3-conjugated secondary antibodies. NeuN was directly labelled with Alexa Fluor 488. For SOX10, sections were incubated with biotinylated secondary antibodies, biotinylated tyramide (Sigma-Aldrich), and streptavidin conjugated with Alexa Fluor 488. Sections were incubated with Hoechst 33342 (Thermo Fisher Scientific).

Brightfield images were taken using the Axioscan Z1 (Zeiss) at 20x magnification. For IF, confocal imaging was performed using a STED microscope (STEDYCON; Abberior Instruments) at 40x magnification. All images were analyzed using QuPath 0.4.0.^59^ Images were excluded if quality was not sufficient for quantification. For example because of weak staining due to probable over-fixation, high amounts of irremovable background or difficulties annotating a lesioned area precisely. Pyramidal tracts were outlined based on anatomical location. Lesions were outlined based on HLA-PLP stains. For SMI312, a thresholder was used. APP^+^ bulbs/axons were manually counted in lesions and NAWM of the pyramidal tract. In mixed lesions, APP^+^ bulbs/axons were normalized to the number of APP^+^ bulbs/axons in surrounding NAWM. A positive cell thresholder was used for quantification of NeuN, CD3, CD79A, DYSF, and ZNF638 stainings. Thresholds were set based on visual inspection. For SOX10, NeuN, ZNF638, and DYSF, positive cells were detected using a random trees-based classifier. NeuN^+^ cells were counted in medial frontal gyrus NAGM or alternatively in fresh frozen sections from the superior temporal gyrus (rs10191329^AA^, *n* = 1; rs10191329^CC^, *n* = 1; no outliers). The number of CD3^+^, a pan T-cell marker, and CD79A^+^, a pan B-cell marker, cells were quantified in lesions and pyramidal tract NAWM. DYSF and ZNF638 were quantified in lesions and in medial frontal gyrus NAWM and NAGM.

#### NfL ELISA

NfL was measured in CSF using the NF-light ELISA CE kit for CSF from Uman Diagnostics (Umea, Sweden, 10-7001). ELISA was performed according to the datasheet. Concentrations were calculated using the standard curve range 50-5,000 pg/ml. Donors with neuropathological signs of a concurrent neurodegenerative disorder or a cerebrovascular accident within a year prior to autopsy were excluded.

#### Tissue RNAseq analysis

Data was adapted from previous work, which isolated white matter lesions for bulk RNA sequencing.^13^ Active lesions and adjacent NAWM were collected from *n* = 96 frozen tissue blocks. RNA was extracted using the RNeasy Micro kit (Qiagen, Hilden, Germany), according to the manufacturer’s instructions. Bulk RNA sequencing was performed on a NovaSeq6000 platform by GenomeScan (Leiden, the Netherlands).

#### Nuclei isolation and RNAseq

Nuclei were isolated from frozen normal appearing super temporal gyrus tissue from the case-control cohort (rs10191329^AA^, *n* = 5; rs10191329^CC^, *n* = 8), according to a previously described protocol.^66^ First, 1mL ice-cold NF1 buffer (10 mM Tris-HCl pH 8.0, 1 mM EDTA, 5 mM MgCl2, 0.1 M sucrose, and 0.5% Triton X-100, 1x proteinase inhibitor) was added to 100 mg of brain tissue and homogenized. Afterwards, the homogenate was incubated in a total volume of 5 mL NF1 buffer for 30 minutes on ice and strained afterwards. Then, the homogenate was diluted in NF1 buffer to a total of 20 mL and a sucrose cushion (1 M sucrose solution with 10 mM Tris-HCl pH8.0, 3 mM MgCl2, and 1 mM DTT) was underlaid. The homogenate was centrifuged at 3,166g for 45 minutes at 4°C without braking, and all supernatant was carefully removed. The nuclei pellet was resuspended in 1 mL NF1 buffer and diluted to a total volume of 10 mL and centrifuged at 1,600g for 5 minutes at 4°C. Then the pellet was resuspended in 10 mL FANS buffer (DPBS, 1% BSA, 1 mM EDTA) and centrifuged 1,600g for 5 minutes at 4°C. The pellet was incubated in FcR blocking reagent for 30 minutes at 4°C and subsequently with Hoechst 33342, rabbit-anti-human-olig2-AF647 (ab225100; Abcam) and mouse-anti-human-NeuN-AF488 (MAB377x; Sigma-Aldrich) for 30 minutes. Single-positive NeuN^+^ or Olig2^+^ nuclei were sorted on a BD FACSAria II cell sorter (BD Biosciences). Total RNA was isolated with the RNeasy MicroKit according to the manufacturers’ instructions. Samples were depleted for rRNA, and DV200 was measured as a quality control on a fragment analyzer (Advanced Analytical, Heidelberg, Germany). Samples were library prepped using the Takara SMARTer Stranded Total RNA-Seq Kit v3 - Pico Input Mammalian Library Prep Kit and sequenced with a depth of 60 million reads by GenomeScan (Leiden, the Netherlands) on an Illumina NovaSeq6000 platform. Visualization of flow cytometry was performed using FlowJo (v10.10.0).

#### Confocal imaging of nuclei

Nuclei of one pilot-sample were isolated as described above, spun down and diluted in mowiol. This was mounted on a glass slide and imaged using a Leica TCS SP8 confocal microscope and Application Suite X (Leica) (Figure S3B). Images were processed using ImageJ (v. 1.54f).

### Quantification and statistical analysis

#### Clinical characteristics

Differences in clinical characteristics were assessed using linear regression, adjusted for sex, age at onset, and initial disease course. Pathological characteristics were tested for differences using a quasi-binomial regression, corrected for sex, age at onset, and initial disease course.

#### Immunohistochemistry

SMI312-positive area, density of DYSF^+^ cells and ZNF638^+^ cells was tested using quasi-binomial regression. APP^+^ bulbs/axons, NeuN^+^ cells, SOX10^+^ cells, CD3^+^ cells, and CD79A^+^ cells per mm^2^ were tested for significance using quasi-Poisson or negative binomial regression, offset by area measured. Statistics were performed in R (v4.3.1) using key packages ggplot2, tidyverse, lme4, glmmTMB, and emmeans. *p*-values <0.05 were considered significant.

#### NfL measurements

We calculated z-scores for NfL using the following formula^67^^,^^68^: CSFNfLz−score=[log2(NfL−value)−(6.661+(age×0.045))]/0.736. Differences between rs10191329^AA^ and rs10191329^CC^ donors were tested using a two-sided t-test. The correlation between proportion of lesions containing foamy macrophages and NfL z-scores were tested using Pearson correlation.

#### Tissue RNAseq

Shortly, Adapter sequences were trimmed with Trimmomatic (v0.39), and alignment was performed against human reference genome GRCh38.105 using default parameters of HISAT2 (v2.2.1). Quality control was performed using FastQC (v0.11.9) and dupRadar (v1.12.1). Counts were obtained using HTseq (v1.99.2) with the ‘Homo_sapiens.GRCh38.105.gtf’ file. Counts were analysed in R (v4.1.0) with Bioconductor (v3.13). Genes with more than two count-per-million reads (CPM) in at least six samples were kept. Count data were normalized using the trimmed mean of M-values (TMM) method^51^ (edgeR package, v3.34.1) and transformed to log2-counts per million (logCPM). Differential gene expression of *DYSF* and *ZNF638* was determined using a t-test.

#### Nuclei RNAseq

Reads were first clipped for adapters using fastp (v0.23.4), and mapped using STAR2 (v0.23.4) using the settings: --outFilterMismatchNmax 2 --outFilterScoreMinOverLread 0 --outFilterMatchNminOverLread 0 --outFilterMatchNmin 60. Separate fastq-files from the same sample were merged after mapping using samtools (v1.20). Quality control was performed using FastQC (v0.12.1). Counts were obtained using featureCounts (v2.0.6) with the ‘gencode.v45.primary_assambly.annotation.gtf’ file. Counts were analyzed in R (v4.3.1). One sample (Olig2 – A:A) was removed, due to having the lowest library size and no olig2 expression. The eventual median library size was 3,2 million counts. Genes with more than two count-per-million reads (CPM) in at least two samples were kept. Count data were transformed to log2-counts per million (logCPM), normalized using the trimmed mean of M-values (TMM) method^51^ (edgeR package, v3.42.4) and precision weighted using voom (limma package, v3.56.2). Remaining genes were reannotated with the biomaRt package (v2.58.2), using Ensembl (v112). PCA was performed on logCPM values of the 500 most variable genes using the PCAtools package (v 2.14.0). For both Olig2^+^ and NeuN^+^ nuclei, the first principal component was strongly correlated with the log10-transformed nuclei number counts and percentage of respectively oligodendrocytes and neurons based on deconvolution (Figure S3C). Therefore, principal component plots were made after correction for this covariate and donor variation, using the function removeBatchEffect (limma package). Proportional abundance of cell types was estimated using marker genes from a single cell study^69^ with dtangle (v2.0.9) deconvolution.

Differential expression was assessed using an empirical Bayes moderated t-test within limma’s linear model framework including estimated proportion of oligodendrocytes or neurons and log10-transformed nucleus number counts as covariates [Y = 0 + experimental condition + estimated cell-type proportion + log10 (cell number)]. *P*-values were corrected for multiple testing using the Benjamini-Hochberg false discovery rate (FDR). Genes with FDR<0.05 were considered significantly differentially expressed. Results were visualized with the ggplot2 package (v3.5.1).

Competitive gene set enrichment analysis was performed with CAMERA with preset value of 0.01 for inter-gene correlation using the Hallmark, C1, C2, C3, C5, C6, and C7 gene set collections from the Molecular Signatures Database (MSigDB, version 2023.2; https://www.gsea-msigdb.org/gsea/msigdb/index.jsp).

## Supplemental information

Document S1. Figures S1–S3

Table S1. Results of differential gene expression analysis and gene set enrichment analysis of bulk nuclei sequencing, related to Figure 4
