# Multiple sclerosis severity variant in *DYSF-ZNF638* locus associates with neuronal loss and inflammation

**DOI:** 10.1016/j.isci.2025.114529

**Published:** 2025-12-26

**Authors:** Hendrik J. Engelenburg, Aletta M.R. van den Bosch, J.Q. Alida Chen, Cheng-Chih Hsiao, Marie-José Melief, Adil Harroud, Inge Huitinga, Jörg Hamann, Joost Smolders

## Main text

(iScience *28*, 112430; May 16, 2025)

Due to errors introduced during the drafting and typesetting of the manuscript, pre-revision versions of Figures 1 and 3 were inadvertently included in the final publication. Additionally, typographical errors were introduced in the title and legend of Figure 4. Finally, incorrect DOI links were provided for references 23, 26, 29, 30, 63, and 69. The authors apologize for these oversights. The figures and text have been corrected in the online version of the manuscript.

**Figure:**
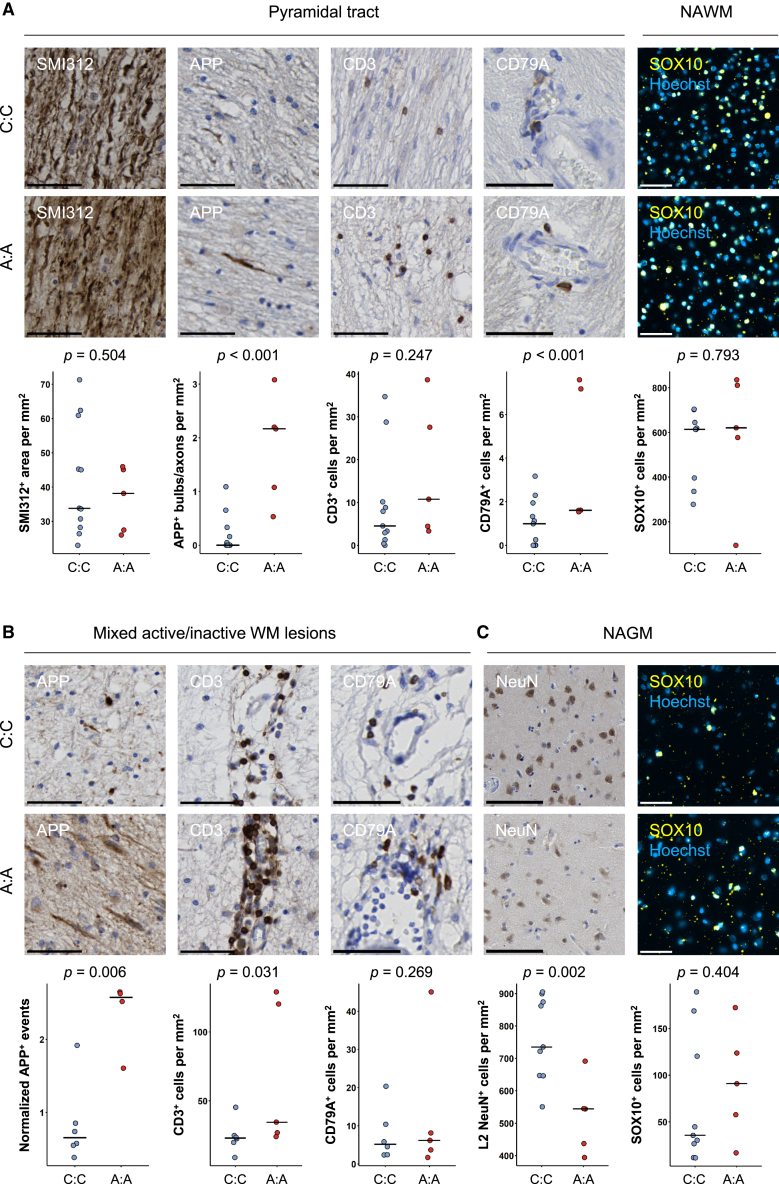
Figure 1. Homozygous carriers of rs10191329^AA^ have more severe neuro-axonal pathology and increased lymphocyte infiltration (corrected)

**Figure:**
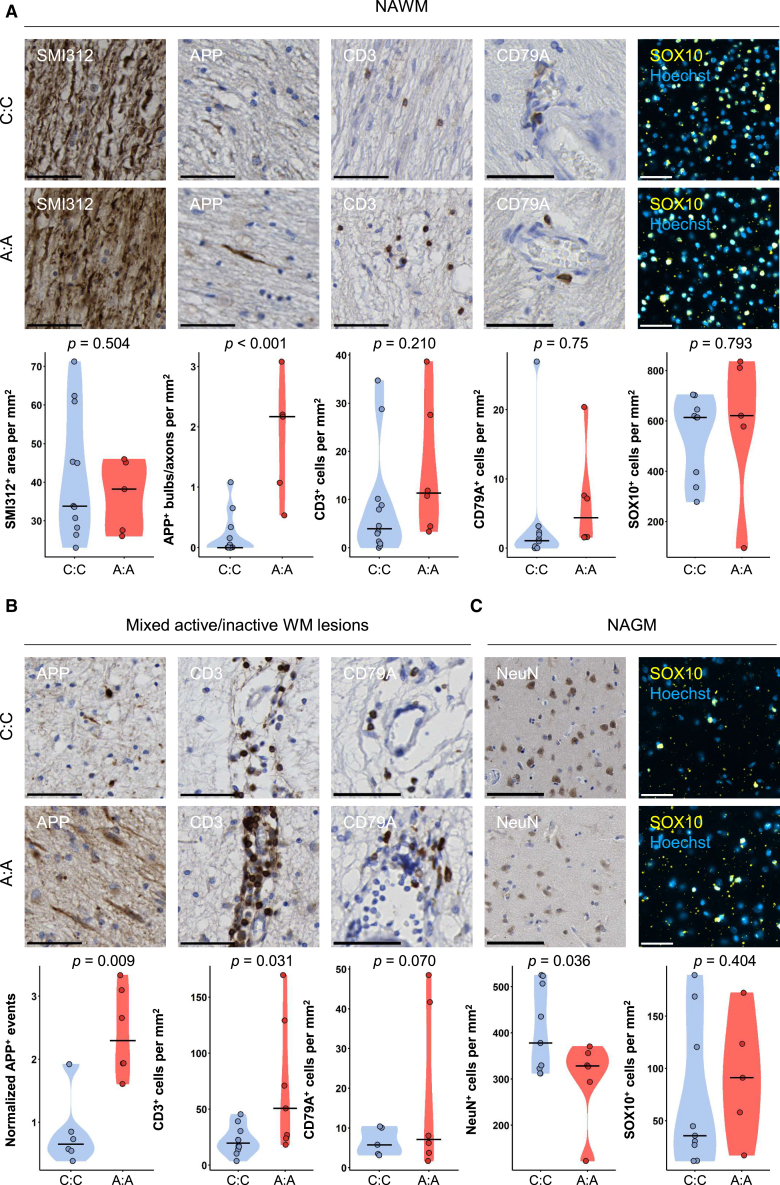
Figure 1. Homozygous carriers of rs10191329^AA^ have more severe neuro-axonal pathology and increased lymphocyte infiltration (original)

**Figure:**
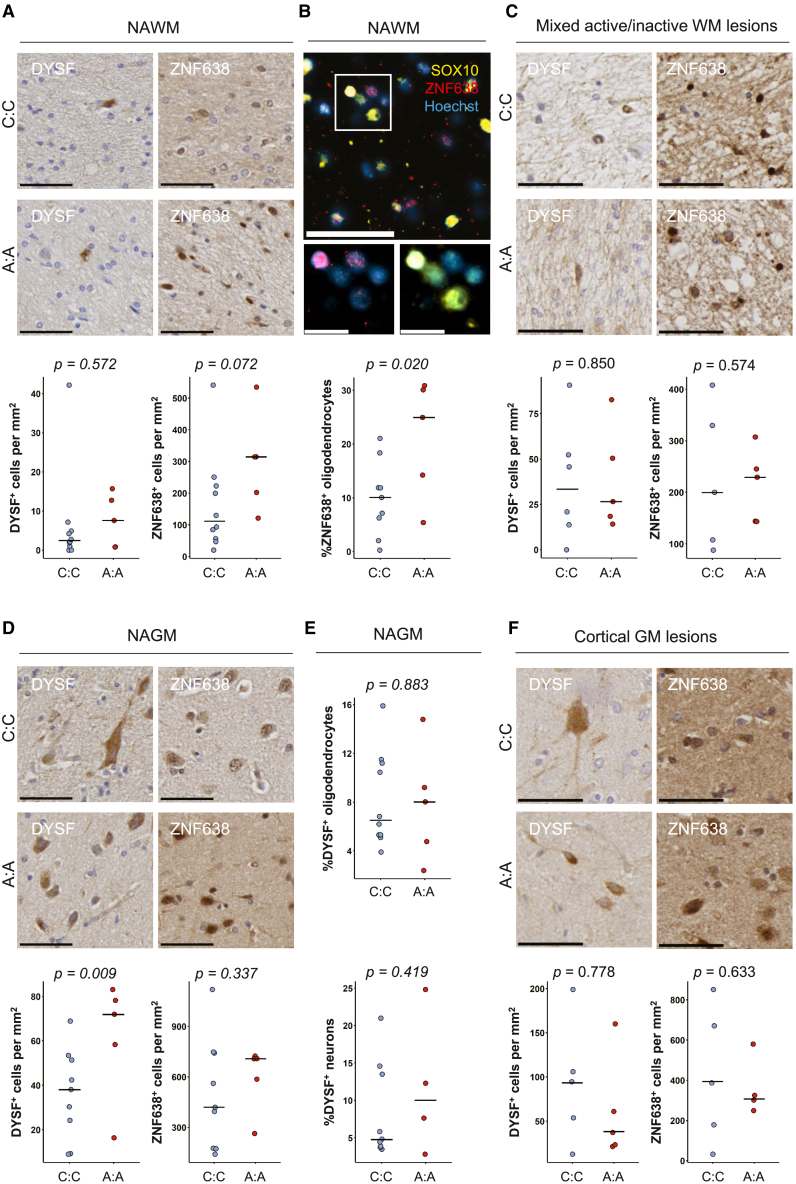
Figure 3. Expression of DYSF and ZNF638 by oligodendrocytes and neurons (corrected)

**Figure:**
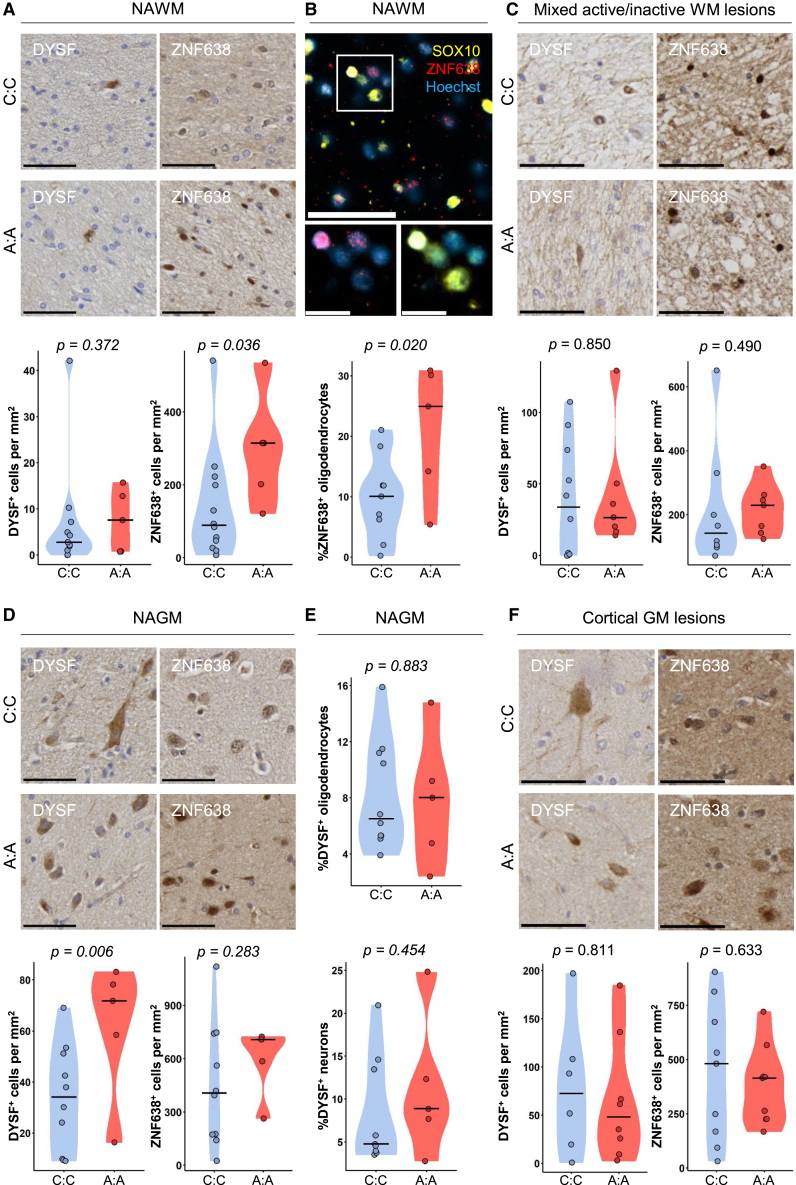
Figure 3. Expression of DYSF and ZNF638 by oligodendrocytes and neurons (original)

