# Supplementary material for: Multiple sclerosis severity variant in DYSF-ZNF638 locus associates with neuronal loss and inflammation
Source: iScience. 2025 Apr 15;28(5):112430. doi: 10.1016/j.isci.2025.112430 (PMC12063138; doi:10.1016/j.isci.2025.112430)
Supplement: Document S1. Figures S1–S3 [file mmc1.pdf]

iScience, Volume *28*

Supplemental information

Multiple sclerosis severity variant in *DYSF-ZNF638* locus associates with neuronal loss and inflammation

Hendrik J. Engelenburg, Aletta M.R. van den Bosch, J.Q. Alida Chen, Cheng-Chih Hsiao, Marie-José Melief, Adil Harroud, Inge Huitinga, Jörg Hamann, and Joost Smolders

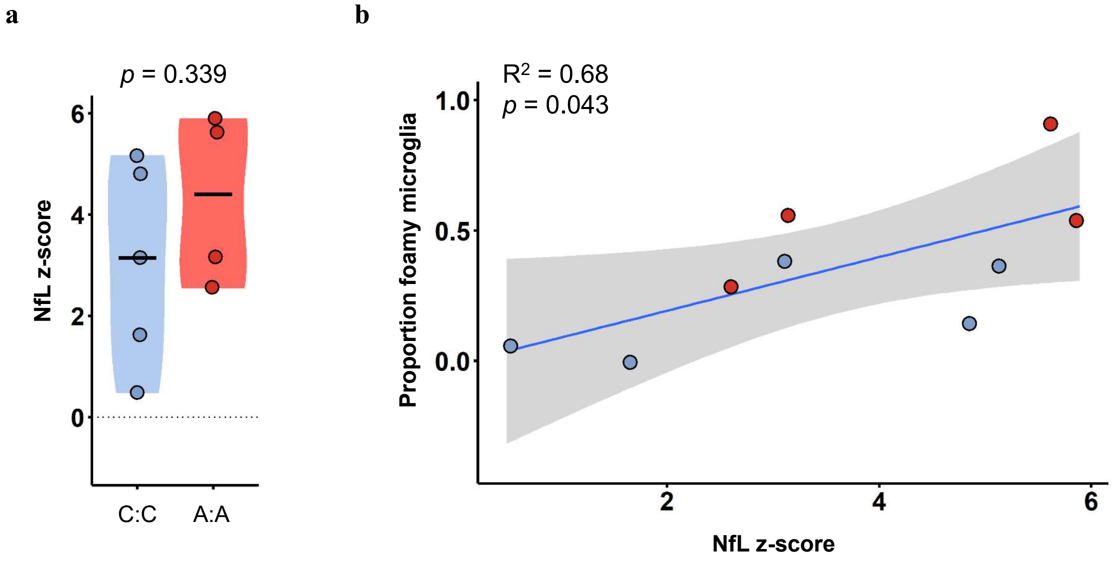

Supplementary Figure 1. NfL correlates with foamy macrophages pathology. Related to Figure 1. **(A)** NfL z-scores not significantly different between rs10191329AA and rs10191329CC donors. Data are represented as individual data points with a bar indicating the mean. Statistics were performed using a two-sided t-test. **(B)** NfL z-scores are significantly correlated with the proportion of (active and mixed) lesions with foamy microglia/macrophage morphology. Statistics were performed using a Pearson correlation. Z-scores were calculated using the formula: $\text{CSF NfL z} - \text{score} = [\log2(\text{NfLvalue}) - (6.661 + (\text{age} \times 0.045))] / 0.736.$ [S1, S2]

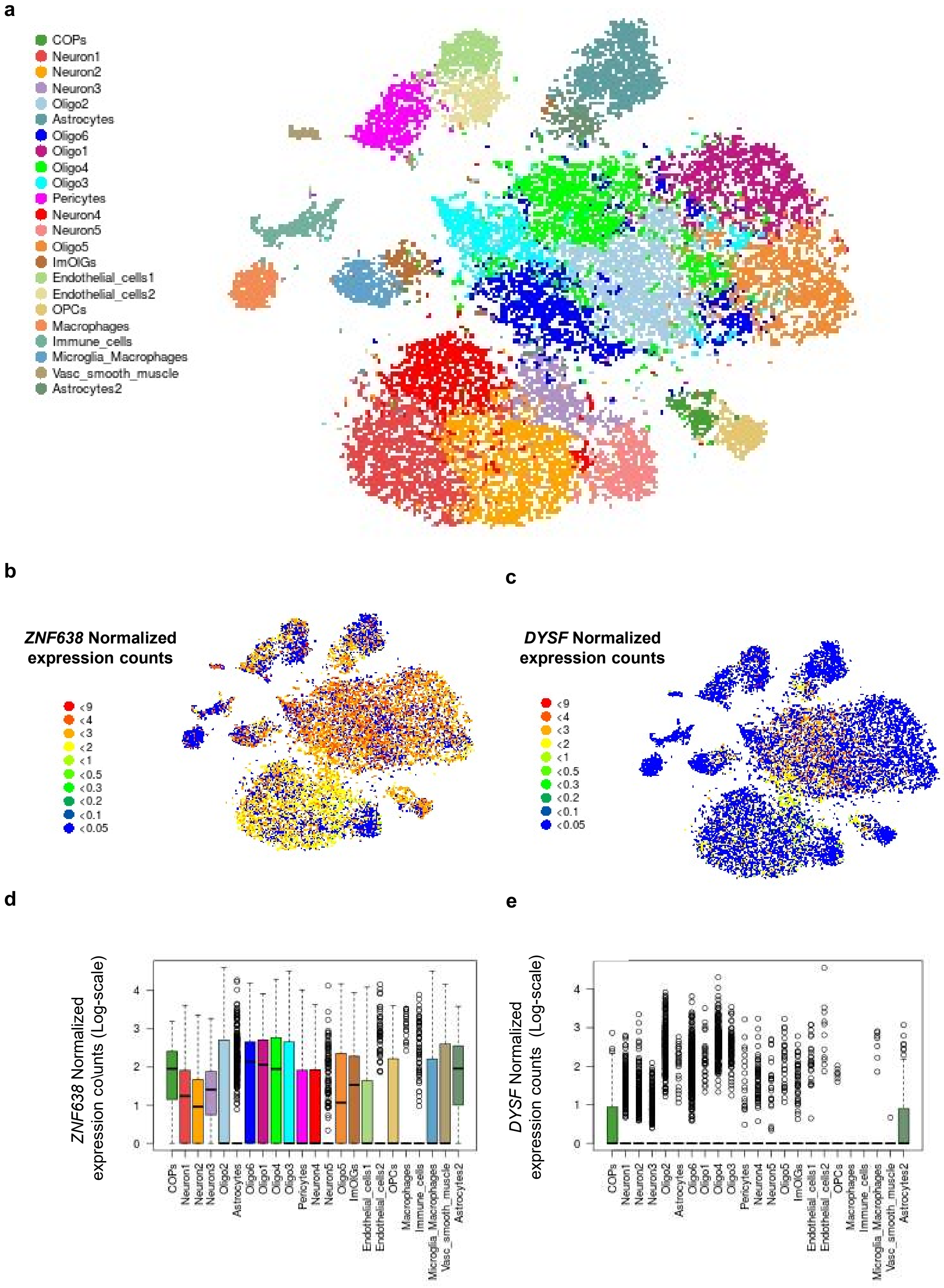

Supplementary Figure 2. *ZNF638* and *DYSF* expression is most abundant in neurons and oligodendrocytes. Related to Figure 2. All data acquired from Jäkel *et al.,* Nature 2019. [S3] **(A)** Single-nuclei tSNE plots colored for cell type. **(B)** *ZNF638* normalized expression counts. **(C)** *DYSF* normalized expression counts. Single-nuclei expression boxplots of **(D)** *ZNF638* normalized expression counts and **(E)** *DYSF* normalized expression counts in different cell type clusters. Data was accessed and plotted via OligoInternode (https://ki.se/en/mbb/oligointernode).

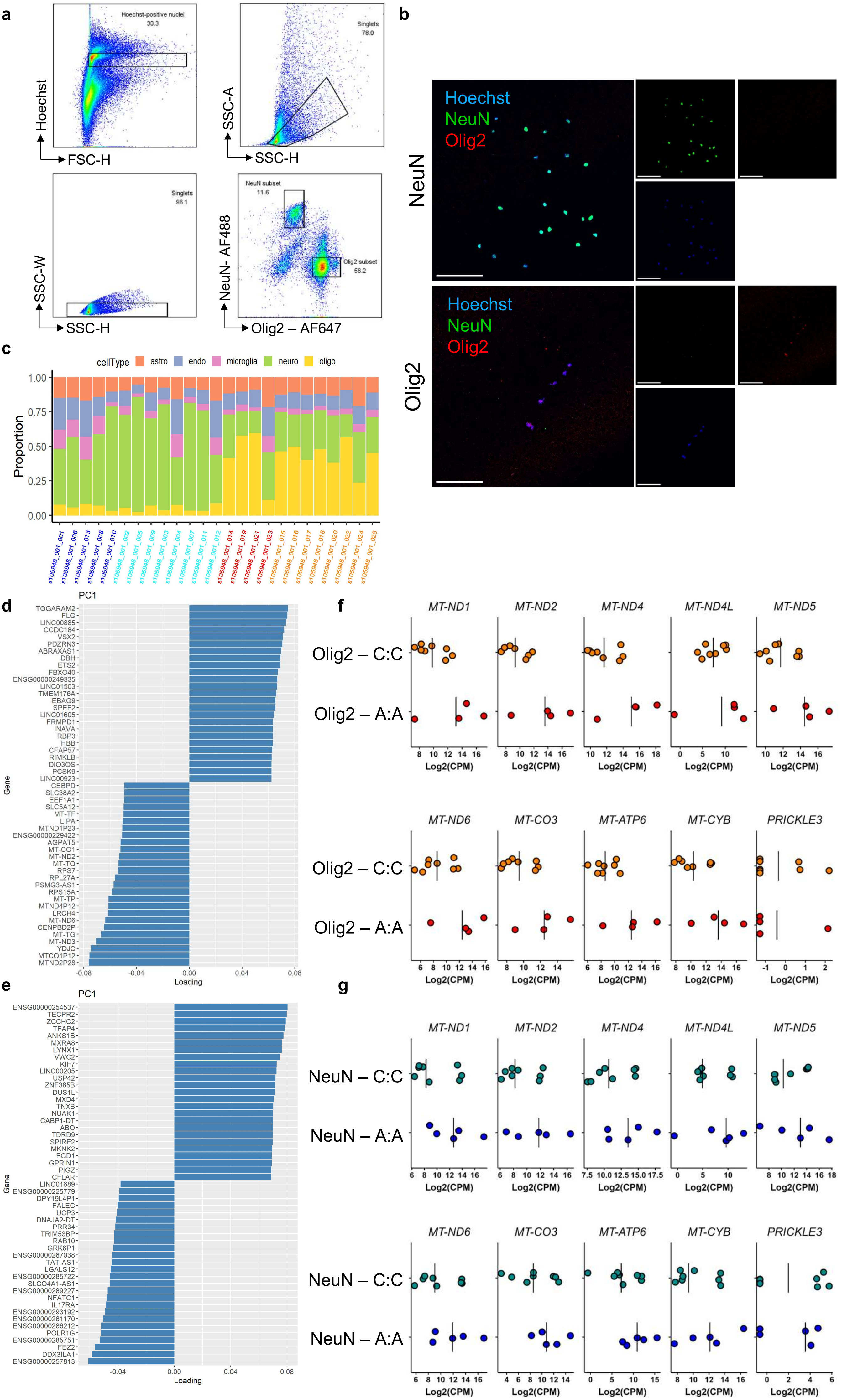
a
Hoechst-positive nuclei
30.3
Hoechst
FSC-H
Singlets
78.0
SSC-A
SSC-H
Singlets
96.1
SSC-W
SSC-H
NeuN subset
11.6
Olig2 subset
56.2
NeuN- AF488
Olig2 – AF647
b
NeuN
Hoechst
NeuN
Olig2
Olig2
Hoechst
NeuN
Olig2
c
cellType
astro
endo
microglia
neuro
oligo
Proportion
1.00
0.75
0.50
0.25
0.00
s105948_001_001
s105948_001_006
s105948_001_013
s105948_001_008
s105948_001_010
s105948_001_002
s105948_001_005
s105948_001_009
s105948_001_003
s105948_001_004
s105948_001_007
s105948_001_011
s105948_001_012
s105948_001_014
s105948_001_019
s105948_001_021
s105948_001_023
s105948_001_015
s105948_001_016
s105948_001_017
s105948_001_018
s105948_001_020
s105948_001_022
s105948_001_024
s105948_001_025
d
PC1
Gene
TOGARAM2
FLG
LINC00885
CCDC184
VSX2
PDZRN3
ABRAXAS1
DBH
ETS2
FBXO40
ENSG00000249335
LINC01503
TMEM176A
EBAG9
SPEF2
LINC01605
FRMPD1
INAVA
RBP3
HBB
CFAP57
RIMKLB
DIO3OS
PCSK9
LINC00923
CEBPD
SLC38A2
EEF1A1
SLC5A12
MT-TF
LIPA
MTND1P23
ENSG00000229422
AGPAT5
MT-CO1
MT-ND2
MT-TQ
RPS7
RPL27A
PSMG3-AS1
RPS15A
MT-TP
MTND4P12
LRCH4
MT-ND6
CENPBD2P
MT-TG
MT-ND3
YDJC
MTCO1P12
MTND2P28
-0.08
-0.04
0.00
0.04
0.08
Loading
e
PC1
Gene
ENSG00000254537
TECPR2
ZCCHC2
TFAP4
ANKS1B
MXRA8
LYNX1
VWC2
KIF7
LINC00205
USP42
ZNF385B
DUS1L
MXD4
TNXB
NUAK1
CABP1-DT
ABO
TDRD9
SPIRE2
MKNK2
FGD1
GPRIN1
PIGZ
CFLAR
LINC01689
ENSG00000225779
DPY19L4P1
FALEC
UCP3
DNAJA2-DT
PRR34
TRIM53BP
RAB10
GRK6P1
ENSG00000287038
TAT-AS1
LGALS12
ENSG00000285722
SLCO4A1-AS1
ENSG00000289227
NFATC1
IL17RA
ENSG00000293192
ENSG00000261170
ENSG00000286212
POLR1G
ENSG00000285751
FEZ2
DDX3ILA1
ENSG00000257813
-0.04
0.00
0.04
0.08
Loading
f
MT-ND1
MT-ND2
MT-ND4
MT-ND4L
MT-ND5
Olig2 – C:C
Olig2 – A:A
Log2(CPM)
MT-ND6
MT-CO3
MT-ATP6
MT-CYB
PRICKLE3
Olig2 – C:C
Olig2 – A:A
Log2(CPM)
g
MT-ND1
MT-ND2
MT-ND4
MT-ND4L
MT-ND5
NeuN – C:C
NeuN – A:A
Log2(CPM)
MT-ND6
MT-CO3
MT-ATP6
MT-CYB
PRICKLE3
NeuN – C:C
NeuN – A:A
Log2(CPM)

Supplementary Figure 3. Mitochondrial genes upregulated in rs10191329^{AA} donors. Related to Figure 4. Bulk RNAseq was performed on isolated nuclei from normal appearing superior temporal gyrus of n = 5 rs10191329^{AA} and n = 8 rs10191329^{CC} donors. **(A)** Representative dot plot showing flow cytometry gating strategy used for isolating nuclear subsets, including singlet gating. **(B)** Fluorescent images of sorted nuclei. Scalebars indicate 100 um. **(C)** Estimated proportional cell type abundance as determined by deconvolution using the dtangle algorithm with the dataset of Darmanis *et al.* [S4] as a reference. Color of the sample name indicate grouping (Blue = NeuN-A:A; Cyan = NeuN-C:C; Red = Olig2-A:A; Orange = Olig2-C:C). **(D)** Loadings of the first principal component of the PCA on Olig2^{+} nuclei. **(E)** Plots of CPMs from Olig2^{+} nuclei of genes in the LHON geneset. **(F)** Loadings of the first principal component of the PCA on NeuN^{+} nuclei. **(E)** Plots of CPMs from NeuN^{+} nuclei of genes in the LHON geneset. CPM = counts-per-million; PC = principal component.

References

[S1] Vermunt, L., Otte, M., Verberk, I.M.W., Killestein, J., Lemstra, A.W., van der Flier, W.M., Pijnenburg, Y.A.L., Vijverberg, E.G.B., Bouwman, F.H., Gravesteijn, G., et al. (2022). Age- and disease-specific reference values for neurofilament light presented in an online interactive support interface. Ann. Clin. Transl. Neurol. *9*, 1832–1837. https://doi.org/10.1002/ACN3.51676.

[S2] Dijkstra, J.I.R., Vermunt, L., Venkatraghavan, V., Ozhegov, G., Coomans, E.M., Ossenkoppele, R., van de Giessen, E., Hulsman, M., de Geus, C.M., van der Flier, W.M., et al. (2025). TREM2 risk variants and associated endophenotypes in alzheimer's disease. Alzheimers. Res. Ther. *17*, 57. https://doi.org/10.1186/S13195-025-01700-2.

[S3] Jäkel, S., Agirre, E., Mendanha Falcão, A., van Bruggen, D., Lee, K.W., Knuesel, I., Malhotra, D., Ffrench-Constant, C., Williams, A., and Castelo-Branco, G. (2019). Altered human oligodendrocyte heterogeneity in multiple sclerosis. Nature *566*, 543–547. https://doi.org/10.1038/s41586-019-0903-2.

[S4] Darmanis, S., Sloan, S.A., Zhang, Y., Enge, M., Caneda, C., Shuer, L.M., Gephart, M.G.H., Barres, B.A., and Quake, S.R. (2015). A survey of human brain transcriptome diversity at the single cell level. Proc. Natl. Acad. Sci. U. S. A. *112*, 7285–7290. https://doi.org/10.1073/PNAS.1507125112/SUPPL_FILE/PNAS.1507125112.SAPP.PDF
